# Clinical Relevance of Oxidative Imbalance in Patients with Temporomandibular Disorder—Myofascial Pain with Referral: An Exploratory Case–Control Study

**DOI:** 10.3390/antiox15091133

**Published:** 2026-09-07

**Authors:** Joanna Kuć, Mateusz Maciejczyk, Krzysztof Dariusz Szarejko, Małgorzata Żendzian-Piotrowska, Walery Tarnawski, Sara Zięba, Anna Zalewska

**Affiliations:** 1 Department of Prosthodontics, Medical University of Bialystok, M. Skłodowskiej-Curie 24A Street, 15-276 Bialystok, Poland; 2 Department of Hygiene, Epidemiology and Environmental Health, Medical University of Bialystok, Mickiewicza 2C Street, 15-022 Bialystok, Poland; mateusz.maciejczyk@umb.edu.pl (M.M.); malgorzata.zendzian-piotrowska@umb.edu.pl (M.Ż.-P.); 3 Private Health Care, Physical Therapy and Rehabilitation, Warszawska 79 Street, 15-201 Bialystok, Poland; krzysztof.sza@poczta.onet.pl; 4 Center for Implantology and Orthodontics “Royal Dental”, Raciborska 69 Street, 44-200 Rybnik, Poland; walery.t@poczta.onet.pl; 5 Independent Researcher, 15-089 Bialystok, Poland; 6 Restorative Dentistry Department, Medical University of Bialystok, M. Skłodowskiej-Curie 24A Street, 15-276 Bialystok, Poland; anna.zalewska1@umb.edu.pl; 7 Independent Laboratory of Experimental Dentistry, Medical University of Bialystok, M. Skłodowskiej-Curie 24A Street, 15-276 Bialystok, Poland

**Keywords:** advanced glycation end products (AGEs), advanced oxidation protein products (AOPPs), antioxidants, myofascial pain with referral, malondialdehyde (MDA), nitric oxide (NO), oxidative stress, oxidative imbalance, temporomandibular disorders (TMDs), thiobarbituric-acid-reactive substances (TBARS)

## Abstract

Background: Temporomandibular disorders constitute multifaced systemic impairments capable of compromising overall physiological homeostasis. This study compares oxidative and nitrosative stress biomarkers between patients with temporomandibular myofascial pain with referral and healthy controls. Methods: We enrolled 44 individuals with temporomandibular myofascial pain with referrals and 44 controls. The procedure involved clinical examination based on the Diagnostic Criteria for Temporomandibular Disorders and saliva collection. Biochemical determination involved advanced glycation end products (AGEs), advanced oxidation protein products (AOPPs), thiobarbituric-acid-reactive substances (TBARS), and total nitric oxide (NO). Results: Median concentrations of TBARS and total NO were significantly elevated in the study group relative to the controls (TBARS: 6.078 vs. 1.486; total NO: 138.6 vs. 23.82), whereas the concentration of AOPPs was significantly lower (151.1 vs. 505.3). Multiple regression revealed that myofascial pain was an independent predictor of the observed differences, even after we adjusted for age and salivary flow rate (TBARS: β = 4.78 [95% CI: 2.020, 7.541], _adj_ *p* = 0.0018; total NO β = 128.4 [95% CI: 82.70, 174.1] _adj_ *p* = 0.0004; AOPP β = −755.7 [95% CI: −1228, −283.5] _adj_ *p*-value = 0.0028. Conclusions: Myofascial pain with referral is independently associated with oxidative imbalance, highlighting the relevance of TBARS and total NO as promising severity biomarkers. The unexpected decrease in AGE and AOPP levels requires standardized studies to confirm diagnostic utility.

## 1. Introduction

Temporomandibular disorders (TMDs) are currently conceptualized as multiple-system dysregulations that, over time, because of their chronicity, trigger an imbalance in the human body’s regulatory framework [1]. According to the literature, only 10–20% of patients with chronic painful TMDs remain free of other pain conditions [1]. Coexisting diseases tend to aggravate over time, leading to central sensitization of pain [1]. In addition, TMD treatment focused solely on masticatory muscles ‘may be misguided at best, and iatrogenic at worst’ [1]. An isolated approach is not merely insufficient; it may inadvertently perpetuate or worsen a patient’s multi-system dysregulation. Quaternary prevention seems to be paramount.

According to the International Classification of Orofacial Pain (ICOP), TMDs and myofascial pain constitute the core components of temporomandibular disorders, representing a major musculoskeletal pain subgroup within the broader spectrum of orofacial pain [2]. Orofacial pain reflects mixed phenotypes, such as nociceptive, neuropathic, neoplastic, and dysfunctional pain, with incompletely understood mechanisms of etiology and pathogenesis [3]. The special roles in these processes may be attributed to oxidative stress levels [3,4]. Oxidative stress is defined as an imbalance between production of reactive oxygen species (ROS) and a decrease in levels of antioxidants responsible for ROS neutralization [5]. Nitrosative stress, which involves biochemical reaction of nitric oxide with the free radical superoxide, is also important in this vein [5]. In orofacial pain, hyperalgesia and allodynia link with increased concentrations of nitric oxide, lipid peroxidation, and mitophagy [3]. The chronicity of orofacial pain is modulated by oxidative stress biomarkers [3]. The kynurenine pathway involved in sensitization of the trigeminal nerve may play an important role in this regard [5]. These implications may indicate that oxidative stress profiles may play an extremely useful role in novel TMD treatment [5,6,7]. Current clinical strategies focus on identification of biomarkers that are involved in painful TMDs, implementation of individually tailored antioxidant treatment, and proper dietary behavior [3,8,9].

In TMDs, the source of oxidative imbalance is loading stress, which leads to the release of free oxygen radicals, arachidonic acid catabolites, neuropeptides, cytokines, and matrix metalloproteinase enzymes [10,11]. These phenomena are conditioned mainly by mechanical pressure within the temporomandibular joint and masticatory muscles [10]. Pathological processes begin at the molecular level and result in hard-tissue destruction. One of the many mechanisms associated with oxidative stress is the hypoxia–reperfusion process modulated by repeated jaw movements during clenching [5]. The consequence is the lubrication system’s collapse, which is recognized as the primary cause of TMDs [10]. Local inflammation and pain promote a cascade of inflammatory reactions within the temporomandibular joint and surrounding muscles [10]. Increases in oxidative metabolism, especially in type I muscle fibers, generate release byproducts that trigger peripheral nociceptor stimulation [10]. Other causes of excessive free-radical formation include direct mechanical injuries, disc derangements, and degenerative changes [5,12]. There is also reason to believe that overuse and/or misuse of the masticatory muscles are involved [5].

Overuse and/or misuse of the masticatory muscles frequently contribute to the development of myofascial pain with referral, which is one of the subtypes of myalgia as per the Diagnostic Criteria for Temporomandibular Disorders (DC/TMD) [13]. This condition is characterized by pain within the temporalis or masseter muscle and pain localized beyond the boundary of the muscle palpated during myofascial examination [13].

While the studies have extensively documented the clinical, epidemiological, and psychosocial aspects of temporomandibular disorders, the precise biochemical alterations—such as oxidative stress—remain less understood. A significant knowledge gap exists regarding the specific biochemical profiles of strictly defined TMD subgroups. This study exclusively focuses on patients diagnosed with myofascial pain with referral. This subgroup represents a more severe and complex clinical phenotype and is strongly associated with greater pain intensity and chronicity, making it an optimal model with which to investigate pronounced biochemical imbalances.

The primary aim of this study was to compare salivary concentrations of selected oxidative and nitrosative stress biomarkers between patients with temporomandibular disorders—myofascial pain with referral—and healthy controls. It was hypothesized that levels of oxidative damage to proteins and lipids, as well as nitrosative stress biomarkers, would be higher in patients with myofascial pain with referral than in the control group.

## 2. Materials and Methods

### 2.1. Ethical Issues

This research was performed upon obtaining the consent of the Bioethics Committee of the Medical University of Bialystok, Poland (permission number—R-I-002/322/2016 and date—29 September 2016, study group sample collection: validity period—1 October 2016–30 December 2017; permission number—APK.002.175.2023, and date—30 March 2023, control group sample collection and biochemical determination: validity period—30 March 2023–1 January 2025; permission number—APK.002.248.2024, and date—18 April 2024, study group biochemical determination: validity period—30 June 2024–31 December 2027), in accordance with the principles of the Declaration of Helsinki of the World Medical Association (WMA) as well as the Guidelines for Good Clinical Practice (GCP). Participation in the study was voluntary. All subjects received comprehensive information about the nature, scope, and course of the clinical procedures. Each subject provided written consent to participate in this research. At each stage of the study, the patients had the right to withdraw from the experiment without any consequences.

### 2.2. Subjects

The research was performed at the Department of Prosthodontics at the Medical University of Bialystok, Poland. The study group consisted of 44 Caucasian patients (32 females, 12 males) with temporomandibular disorder—that is, myofascial pain with referral. The average age was 23.6 ± 1.9, and the age range was 18 to 25 years. The subjects were diagnosed with respect to the Diagnostic Criteria for Temporomandibular Disorders (DC/TMD) [14]. Clinical examination was performed by an experienced dentist specializing in prosthodontics and a physiotherapist (J.K., one of the authors) for one person. The patients were originally recruited from the Department of Prosthodontics at the Medical University of Bialystok, Poland, representing a group from the same geographical area.

The study group included a specifically selected subset of individuals from our broader selected cohort [15]. The pain pattern of this study group has been previously described in detail [15]. Briefly, to confirm the chronicity of the condition, patients were evaluated using the Graded Chronic Pain Scale (GCPS v.2.0), which assesses pain over the preceding 6 months. As previously reported, the patients exhibited varying degrees of chronic-pain severity [15]. Since, for this study group, we analyzed a subset of the broader cohort, the distribution of chronic-pain severity in this study group was as follows:

- Study group (n = 44): Grade 0 (6.82%), Grade I (61.36%), Grade II (11.36%), Grade III (11.36%), and Grade IV (9.09%).
- Subgroup of women (n = 32): Grade 0 (6.25%), Grade I (68.75%), Grade II (9.38%), Grade III (3.13%), and Grade IV (12.5%).
- Subgroup of men (n = 12): Grade 0 (8.13%), Grade I (41.7%), Grade II (16.7%), Grade III (33.3%), and Grade IV (0%).

**Inclusion criteria**

- Having a confirmed diagnosis of myofascial pain with referral with respect to DC/TMD;
- Having pain located in the craniofacial and/or craniomandibular area (VAS ≥ 8) on the day of the clinical examination;
- Having complete natural dentition with class I of Angle’s Molar Classification and proper canine alignment, all of which constitute the defining features of optimal occlusion;
- Having no history of corrective orthodontic procedures or retention status above 36 months after completion of tooth realignment therapy.

**Exclusion criteria**

- Having a history of trauma within the craniofacial and/or craniomandibular area;
- Having undergone any surgical intervention within the craniofacial and/or craniomandibular region (e.g., orthognathic surgery, otolaryngologic procedures, ophthalmic surgery, plastic surgery, and dental implants);
- Having previously undergone occlusal splint therapy or currently being treated with it;
- Having a history of prosthodontic treatment (e.g., dental crowns, veneers and bridges, removable prostheses, and dental implants);
- Having previously undergone or currently undergoing physiotherapy within the craniofacial and/or craniomandibular region (e.g., myofascial release, trigger-point therapy, dry needling, and temporomandibular joint mobilization);
- Being afflicted with any diseases affecting the function of the masticatory muscles (e.g., fibromyalgia, rheumatoid arthritis, polymyalgia, multiple sclerosis, and Lyme disease);
- Having any metabolic disorders (e.g., diabetes, metabolic syndrome, thyroid disorders);
- Taking or having taken any medications, including chronic drug therapy, (e.g., antidepressants, steroids, hormone therapy, chemotherapy);
- Having adhered to any specific diets or taken any supplements within the past 6 months (e.g., the Mediterranean diet; anti-inflammatory diets; intermittent fasting; multivitamin complexes; and supplementation with any form of vitamins B, C, or E; coenzyme Q10; or minerals such as magnesium, iron, and potassium) [9]

Such restrictive criteria were a deliberate methodological choice reflecting an exploratory design aimed at eliminating potential confounding factors that could alter salivary and oxidative stress biomarker concentrations. Because salivary and oxidative stress biomarkers are highly sensitive to local and systemic confounders, strict sample homogeneity was prioritized to ensure high internal validity.

The control group comprised 44 healthy Caucasian participants (22 females, 22 males) aged 18–25, with a mean age of 22.3 ± 2.2. They were originally recruited from the Department of Conservative Dentistry at the Medical University of Bialystok, Poland, constituting a group from the same geographical area. All participants were free of pain in the craniofacial and craniomandibular regions, including referred myofascial pain. In addition, all control subjects met the predefined exclusion criteria.

The participants included in this study are identical to those described in our earlier publication [9]. Consequently, their clinical characteristics and salivary flow rates have already been published [9]. However, all the biochemical analyses evaluating products of oxidative damage to proteins and lipids, as well as nitrosative stress biomarkers, are entirely novel, having been conducted specifically for the purposes of this manuscript, and have not been reported elsewhere.

### 2.3. Saliva Collection

The spitting method was used to collect non stimulated saliva. The material was obtained after an overnight rest, and collection was always carried out between 8.00 and 9.00 a.m. The patients did not consume any food and drinks, with the exception of pure water, which was drunk at least 2 h before saliva was obtained. All hygiene procedures applicable to the oral cavity were also discontinued. To ensure that there were comfortable, non-stressful conditions, the saliva was collected at the Department of Prosthodontics at the Medical University of Bialystok, Poland, in the same room where the clinical examination was conducted. Sampling was preceded by rinsing of the mouth twice with distilled water at room temperature and at least 5 min of adaptation to environmental conditions. The patients were directed to sit in a dental chair, tilt their heads slightly downwards, and limit movement of the face and mouth. Secreted saliva was collected directly into sterile Falcon^®^ tubes (BD Biosciences, San Jose, CA, USA) and then placed in an ice bucket. The collection time was 10 min, with a maximum volume of 5 mL, and any saliva spat out in the first minute was omitted. This method was described in another study [16].

A calibrated pipette with an accuracy of 100 μL was used to determine the volume of saliva, and the flow of non-stimulated saliva was obtained by dividing the volume of saliva by the time taken for secretion. After sampling, the saliva was immediately centrifuged under constant conditions (20 min., 3000× *g*, +4 °C, MPW 35; MPW Med. Instruments, Warsaw, Poland). To protect the samples from oxidation due to their processing and storage, butylated hydroxytoluene was added to the supernatants (BHT, Sigma-Aldrich, Saint Louis, MO, USA; 10 μL of 0.5M BHT per 1mL of saliva) [16]. For biochemical tests, saliva samples were frozen at −80 °C and then stored under these conditions until analysis. The samples did not undergo any freeze–thaw cycles prior to biochemical analysis.

### 2.4. Biochemical Determination

Biochemical analyses included determination of

- The products of oxidative damage to proteins, namely, advanced glycation end products (AGEs) and advanced oxidation protein products (AOPPs);
- The products of oxidative damage to lipids, i.e., thiobarbituric-acid-reactive substances (TBARS);
- Nitrosative stress, i.e., levels of nitric oxide (NO).

All determinations were made using non-stimulated saliva. The material was slowly thawed at 4° on the day of the assays. All reagents were sourced from Sigma-Aldrich Company (Numbrecht, Germany/Saint Louis, MO, USA). To evaluate the absorbance/fluorescence of the samples, a 96-well microplate reader was used (Infinite M200 PRO Multimode Microplate Reader Tecan; Tecan Group Ltd. Mannedorf, Switzerland). The results were standardized with respect to 1 mg of total protein.

The total protein concentration was determined using the bicinchoninic acid (BCA) assay [17] via the Pierce BCA Protein Assay Kit (Thermo Fisher Scientific, Rockford, IL, USA), with bovine serum albumin (BSA) serving as the calibration standard. The assessment was conducted spectrophotometrically at a wavelength of 562 nm. The concentration of total protein was expressed in μg/mL.

#### 2.4.1. Products of Oxidative Damage to Proteins and Lipids

The concentration of advanced glycation end products (AGEs) was determined using the spectrofluorimetric method. The measurement is based on the fluorescence of pentosidine, pyraline, carboxymethyl lysine (CML), and furyl-furanyl-imidazole (FFI) at a wavelength 350/440 in duplicate samples. Before biochemical determination, the saliva samples were diluted in 0.1 M H_2_SO_4_ (1:5, *v*/*v*). The results are expressed in arbitrary fluorescence units (AFU)/mg of protein [18,19].

Advanced oxidation protein product (AOPP) content was determined using the colorimetric method in duplicate samples. The measurement was based on the oxidation capacity of iodine ions at a 340 nm wavelength [18].

The concentration of thiobarbituric-acid-reactive substances (TBARS) was established via the spectrophotometric method using thiobarbituric acid (TBA). Briefly, 1.0 mL of each saliva sample was combined with 2.0 mL of TCA-TBA-HCL and mixed thoroughly [20]. The solution was incubated for 15 min in boiling water and then rapidly cooled on ice to stop the reaction. To separate the resulting flocculent precipitate, the cooled mixture was centrifuged (for 10 min at 1000× *g*) [20]. The absorbance of the samples was measured at 535 nm using a 96-well microplate reader, and 1,1′,3,3′-tetrahydroxypropane was applied as a standard [20].

#### 2.4.2. Nitrosative Stress Parameters

Total nitric oxide (NO) content was determined via the colorimetric method, including sulfanilamide and NEDA 2 HCL (N-(1-naphthyl)-ethylenediamine dihydrochloride). Nitrate reductase was applied to transform nitrate into nitrite. The concentration of total NO was spectrophotometrically measured at a wavelength of 490 nm and expressed in μmol/mg protein [21].

### 2.5. Statistical Analysis

Data were analyzed using Graph Pad Prism 8 software (GraphPad Software, La Jolla, CA, USA). Normality of distribution was assessed with the Shapiro–Wilk test. Median values were used to describe the central tendency of individual biomarker levels, while arithmetic means and standard deviations were calculated to summarize the distribution of the data.

To compare salivary concentrations of biomarkers between the study and control groups while adjusting for potential confounders, multiple-regression analyses were performed. In light of the significant disparity in the gender distribution between the groups and to reveal potential differences, a stratified analytical approach was employed. For each biomarker, three separate regression models were proposed. We applied the primary model to the entire cohort (n = 88), adjusting for gender, age, and salivary flow rate. Subsequently, we created separate gender-stratified models for all men (n = 34) and all women (n = 54), adjusting only for age and salivary flow rate. The differences were presented as effect sizes, demonstrated by adjusted unstandardized regression coefficients (β) with corresponding 95% confidence intervals (95% CIs). To control the false-positive rate across the multiple testing of the four selected biomarkers, *p*-values were corrected using the Benjamini–Hochberg false-discovery-rate (FDR) procedure with a threshold of Q = 0.05. To avoid making overly conservative corrections across different clinical hypotheses, FDR adjustment was performed independently within each analytical family; it was employed separately for the cohort group, the male subgroup, and the female subgroup.

Interdependence between salivary parameters was assessed using Spearman’s rank correlation coefficient (r). The derived correlation matrix was graphically rendered as a heatmap. To strictly control the false-discovery rate (FDR) associated with multiple comparisons, raw *p*-values were corrected using the Benjamini–Hochberg procedure. Statistical significance was established at an adjusted *p*-value (FDR q-value) threshold <0.05.

## 3. Results

We enrolled a total of 44 Caucasian patients (32 women, 12 men) with temporomandibular myofascial pain with referral. The subjects ranged in age from 18 to 25, with an average of 22.3 ± 2.2. The control group comprised 44 healthy Caucasian subjects (22 women, 22 men) who ranged in age from 18 to 25, with a mean age of 22.3 ± 2.2.

**Figure 1:**
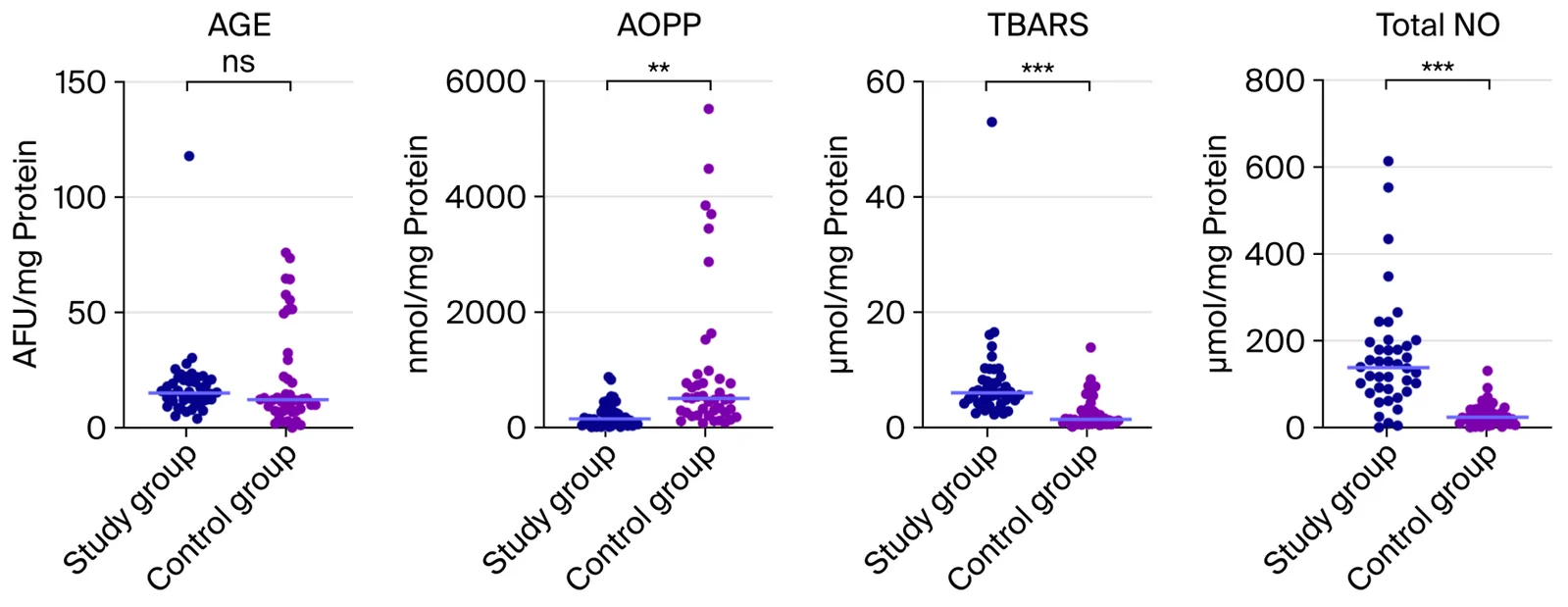
Concentrations of advanced glycation end products (AGEs), advanced oxidation protein products (AOPPs), products of oxidative damage to lipids (thiobarbituric-acid-reactive substances (TBARS)), and total nitric oxide (NO) in non-stimulated saliva in the study group (n = 44) and control group (n = 44). Individual datapoints are shown as dots, with the horizontal line indicating the median. Group comparisons were performed using multiple linear regression adjusted for age and salivary flow rate. *p*-values were adjusted for multiple comparisons using the Benjamini–Hochberg FDR method, yielding the reported _adj_ *p*-values (** < 0.01, *** < 0.001). Note: ns—non significant.

We found statistically significant differences in salivary concentrations of AOPPs, TBARS, and NO between the patients suffering from temporomandibular disorders and the healthy controls (*p* < 0.05) (Table 1) (Figure 1).

Higher concentrations of TBARS and total NO were found in the patients with temporomandibular disorder, namely, myofascial pain with referral (Me _TBARS Study group_ = 6.078, and Me _TBARS Control group_ = 1.486; Me _Total NO Study group_ = 138.6, and Me _Total NO Control group_ = 23.82) (Table 1) (Figure 1). Multiple linear regression confirmed that TBARS (β = 4.78; 95% CI:2.02, 7.541; _adj_ *p* = 0.0018) and total NO (β = 128.4; 95% CI:82.70, 174.1; _adj_ *p* < 0.0004) were significantly associated with myofascial pain with referral (Table 1) (Figure 1).

Statistically higher concentrations of AOPPs were observed in the control group (Me _AOPP Study group_ = 151.1; Me _AOPP Control group_ = 505.3) (Table 1) (Figure 1). Multiple linear regression revealed that a low concentration of AOPPs was significantly associated with myofascial pain with referral (β = −755.7; 95% CI: −1228, −283.5; _adj_ *p* = 0.0028) (Table 1) (Figure 1). With respect to AGEs, the differences were not statistically significant between the groups (β = −5.35; 95% CI: −14.34,3.644; _adj_ *p* = 0.2403) (Table 1) (Figure 1).

**Figure 2:**
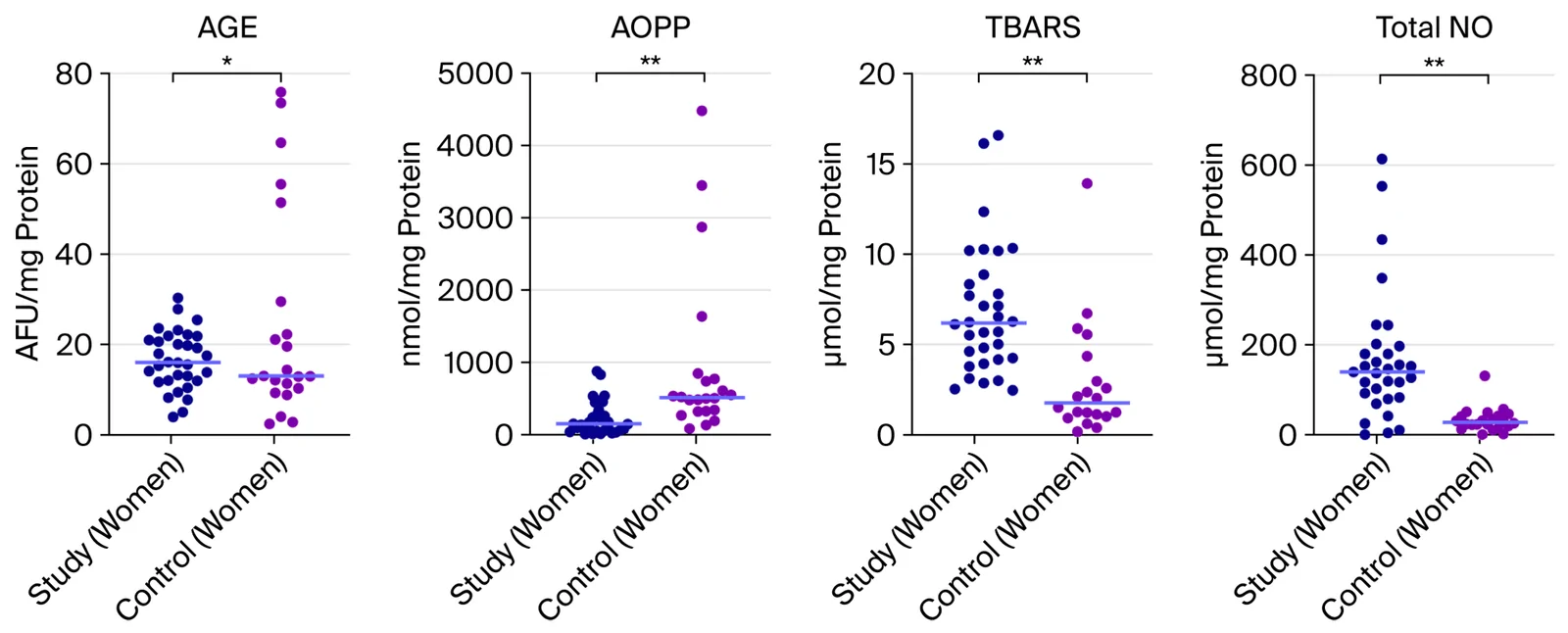
Concentrations of advanced glycation end products (AGE), advanced oxidation protein products (AOPP), products of oxidative damage to lipids (thiobarbituric-acid-reactive substances (TBARS)), and total nitric oxide (NO) in non-stimulated saliva in the female study group (n = 32) and female control group (n = 22). Individual datapoints are shown as dots, with the horizontal line indicating the median. Group comparisons were performed using multiple linear regression adjusted for age and salivary flow rate. *p*-values were adjusted for multiple comparisons using the Benjamini–Hochberg FDR method, yielding the reported _adj_ *p*-values (* < 0.05, ** < 0.01).

**Figure 3:**
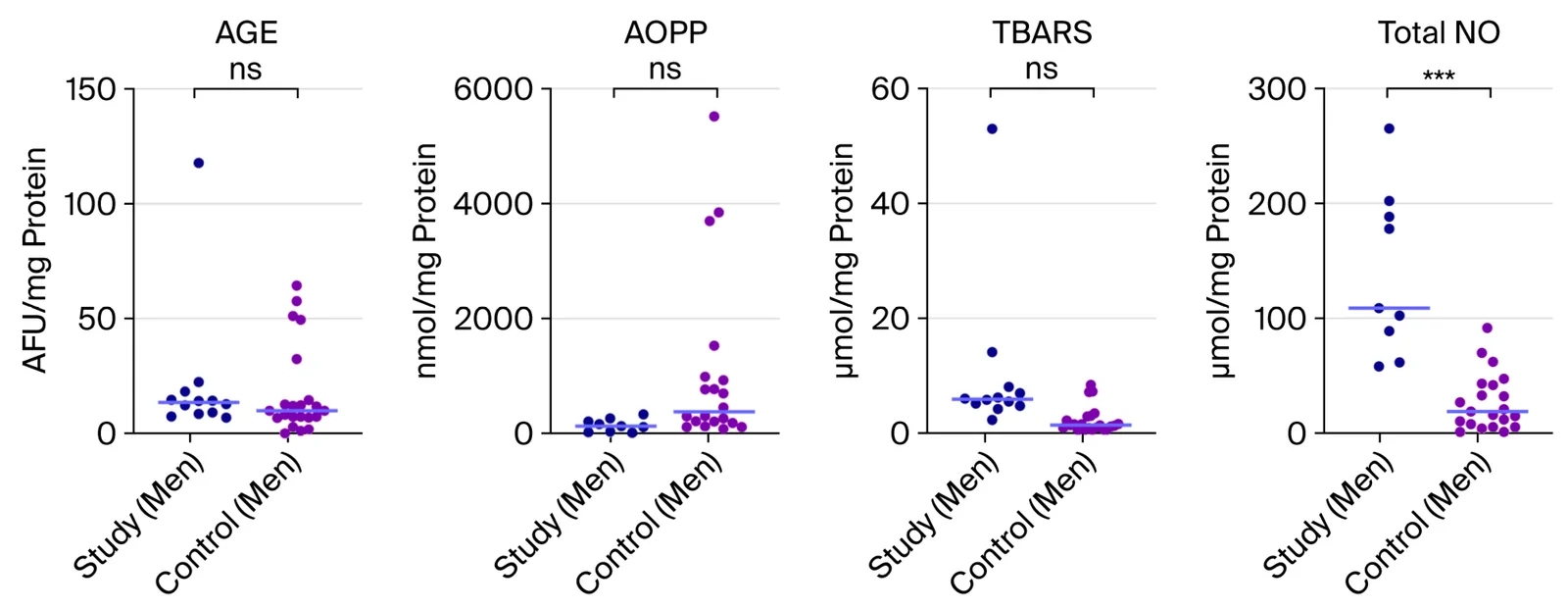
Concentrations of advanced glycation end products (AGEs), advanced oxidation protein products (AOPPs), products of oxidative damage to lipids (thiobarbituric-acid-reactive substances (TBARS)), and total nitric oxide (NO) in non-stimulated saliva in the male study group (n = 12) and male control group (n = 22). Individual datapoints are shown as dots, with the horizontal line indicating the median. Group comparisons were performed using multiple linear regression adjusted for age and salivary flow rate. *p*-values were adjusted for multiple comparisons using the Benjamini–Hochberg FDR method, yielding the reported _adj_ *p*-values (*** < 0.001). Note: ns—non significant.

With respect to gender, women and men with temporomandibular disorder had statistically significantly higher concentrations of TBARS and total NO relative to the relevant healthy controls (Me _TBARS Study group of women_ = 6.184, and Me _TBARS Control group of women_ = 1.781; Me _TBARS Study group of men_ = 5.932, and Me _TBARS Control group of men_ = 1.405; Me _Total NO Study group of women_ = 140, and Me _Total NO Control group of women_ = 27.63; Me _Total NO Study group of men_ = 109.2, and Me _Total NO Control group of men_ = 19.22) (Table 1) (Figure 2 and Figure 3).

Multiple linear regression revealed that only in women were elevated levels of TBARS significantly associated with myofascial pain with referral (β = 3.36; 95% CI: 1.317, 5.393; _adj_ *p* = 0.0024) (Table 1) (Figure 2 and Figure 3).

In the case of total NO, statistically significantly higher values were observed in both women (β = 123.7; 95% CI: 55.91, 191.5; _adj_ *p* = 0.0024) and men (β = 116.2; 95% CI: 70.66, 161.7; _adj_ *p* < 0.0004) with myofascial pain with referral (Table 1) (Figure 2 and Figure 3).

With respect to AGEs, multiple linear regression revealed statistically significant differences only in women (β = −11.17; 95% CI: −20.30, 2.046; _adj_ *p* = 0.0174) (Table 1) (Figure 2 and Figure 3).

The salivary flow measurements were previously published in Ref [9]. As previously reported for this cohort, the study group demonstrated a significantly higher salivary flow rate relative to the healthy controls (median = 0.550 vs. 0.400) [9]. This pattern remained consistent among female participants when evaluated against their control counterparts (median = 0.600 vs. 0.400) [9]. Conversely, no significant variation in flow rate was identified between the male participants (*p* > 0.05) [9]. These previously published salivary flow data provide necessary physiological context and clinical background for our new findings in this manuscript.

**Figure 4:**
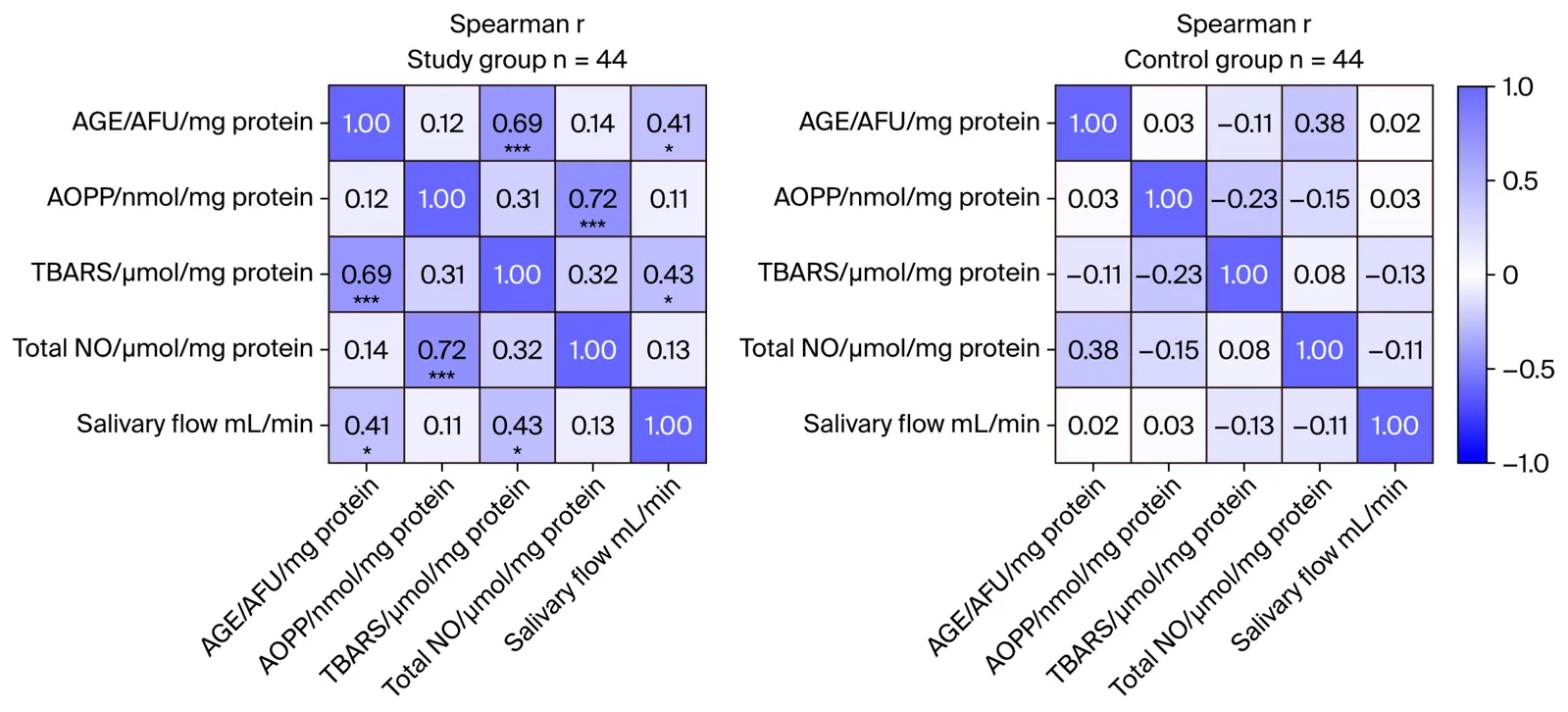
Heatmap correlations between salivary AGEs, AOPPs, TBARS, total NO, and salivary flow rate in the study group (n = 44) and control group (n = 44). The color gradient and the values inside the cells indicate the strength and direction of Spearman’s correlation coefficients (r). To account for multiple testing, nominal *p*-values were adjusted using the Benjamini–Hochberg false-discovery rate (FDR) procedure. Asterisks indicate statistically significant correlations based on the FDR-adjusted *p*-value * < 0.05, *** < 0.001.

Following FDR adjustment, a directly proportional correlation was observed between the salivary flow rate and the salivary concentrations of AGEs and TBARS in individuals presenting myofascial pain with referral (r = 0.406, _adj_ *p* = 0.015; r = 0.430, _adj_ *p* = 0.013, respectively) (Figure 4). A statistically significant relationship was found between AOPPs and total NO (r = 0.72, _adj_ *p* = 0.0000014) and between TBARS and AGEs (r = 0.69, _adj_ *p* = 0.0000014) (Figure 4). No statistically significant correlations between the biomarkers investigated were observed in the control group (Figure 4).

## 4. Discussion

This study’s findings provide novel insights into temporomandibular disorders, highlighting the importance of an integrated approach that considers the complex interactions between myofascial pain, oxidative and nitrosative biomarkers, and systematic regulatory pathways. Despite oxidative stress’s well-established involvement in human diseases, its contribution to the pathophysiology of temporomandibular disorders remains insufficiently characterized. Recent clinical and experimental evidence suggests that assessment of oxidative and nitrosative stress biomarkers may improve our understanding of TMD pathogenesis and facilitate the development of individualized therapeutic strategies.

Among the biomarkers investigated in this study, advanced glycation end products (AGEs) have emerged as being particularly noteworthy. AGEs are heterogeneous molecules that initially arise through non-enzymatic reactions based on glycation/condensation processes between reducing sugars, such as glucose or fructose, and the free amino groups of proteins, lipids, and nucleic acids [22]. These interactions lead to the formation of highly reversible Schiff bases, which are subsequently transformed into more stable Amadori products [22]. Amadori products are converted into covalent adducts and accumulate on proteins. However, intermediate Amadori products may be reversible [22]. The above processes reflect the Maillard reaction, in which the Hodge pathway generates extremely responsive dicarbonyls (α-oxoaldehydes) such as methylglyoxal, glyoxal, and deoxyglucosone. Dicarbonyls promote the synthesis of advanced glycation end products [22]. There are other routes of AGE creation aside from the Maillard reaction. Alternative pathways include the Namiki route, which is based on the splitting of dicarbonyl compounds from aldimines; the Wolff pathway, which is referred to as metal-catalyzed glucose autooxidation; glycolytic pathway intermediates; the polyol route, concerning sorbitol conversion; lipid peroxidation; and ketone body metabolism [22,23,24].

In the human body, AGEs accumulate over the course of physiological senescence and age-related disorders [25]. They play significant roles in skeletal muscle aging. The consequence of AGEs’ aggregation is neuromusculoskeletal degradation, wherein these molecules disrupt the biomechanical properties of the tissues [25]. Pathological changes may concern bone, cartilage, muscles, tendons, ligaments, and nerves [26]. All these structures make up the stomatognathic system, and, with this in mind, age-related changes should be also considered. Modification of proteins under the influence of AGEs leads to structural conversion by altering the extracellular matrix owing to charge changes and the creation of cross-linkages [26]. Non-enzymatic, non-physiological cross-links promote random and disorderly binding of molecules within collagens, and their generation depends on the degree of glycemia and oxidation [26]. The increase in AGE concentrations is accelerated in subjects with diabetes and during the aging process. In the former case, hyperglycemia promotes AGE formation [27]. In the latter, the cause is the imbalance between AGE production and the clearance of AGE accumulation. Previous studies have revealed that the annual AGE accumulation rate amounts to about 3.7% in early adulthood, with this figure increasing to 33% by age 80 [27].

Importantly, the binding of AGEs to the appropriate RAGE receptor triggers inflammation and oxidative stress via intracellular signal transduction [26]. Activation of NADPH oxidase boosts ROS production with relevant ROS-related AGE creation [22]. In addition, ROS are implicated in inflammatory responses and RAGE upregulation [22]. This dependence creates a kind of a positive feedback loop [22]. AGE formation may result in musculoskeletal disorders such as osteoporosis, osteoarthritis, neuropathy, tendinopathy, ligament inflammation (hypertrophy and ossification), and tendinopathy [26]. Any changes in the intramuscular connective tissue due to AGE cross-linking may modify the function of the peripheral nerve and the velocity of muscle innervation [28].

An in vitro study indicated that increased AGE cross-linking may contribute to a reduction in the toughness of cortical and cancellous bone [26]. At the same time, a study on mice revealed ~8.3×-higher accumulation of the advanced glycation end product carboxymethyl-lysine (CML) in the mandible relative to the femur [29]. Unlike the femur, the mandible demonstrated higher collagen maturation and mineral crystallinity, a greater Young’s modulus, and lower carbonation [29]. Therefore, the importance of AGEs in the context of bone structure and dental implantation remains to be elucidated. More research is needed.

AGE cross-linking leads to an increase in cartilage collagen stiffness, subsequently contributing to changes in biomechanical properties [26]. This phenomenon is gaining importance in relation to the mechanical stiffness of TMJ condylar cartilage [30]. A key aspect involves alterations in collagen cross-linking that affect fiber–fiber and fibril–fibril sliding. These phenomena may determine the mechanical responses of TMJ condylar cartilage [30]. Composed mainly of type I collagen, the TMJ layer is considered a key structural component responsible for resistance to compression and the ability to withstand frictional forces during temporomandibular joint function [30]. Cartilage collagen displays an extremely low turnover rate and remains largely unreplaced after maturation. This limited renewal promotes gradual accumulation of AGEs in joint tissue throughout the aging process [30].

With respect to skeletal muscles, by binding to RAGE on the membranes of skeletal muscle cells, AGEs trigger inflammation and boost nicotinamide adenine dinucleotide phosphate oxidase (NADPH oxidase) levels, thus increasing the concentration of circulating reactive oxygen species [26]. The consequence is acceleration of the degradation of muscle proteins and endothelial dysfunction in the skeletal muscle microcirculation [25,26]. Considering the pathophysiology of myofascial pain with referral, microcirculation disorders probably lead to a delay in the discharge of metabolic products deposited in the muscle tissue. This may be exacerbated by persistent muscle tension and contribute to the persistence of pain. Notably, muscle mass reduces by about 1% per year starting at age 25, and women have less muscle mass than men [31]. Muscle loss accelerates after the age of 50, with skeletal muscle mass decreasing by approximately 1–2% annually and reaching 3–5% per year in the elderly [32]. From the age of 40–80, about 30–50% may completely disappear [32]. AGEs accelerate this process [25]. Furthermore, skeletal muscles constitute approximately half of a human’s total bodyweight. Interestingly, about 70% of dietary glucose intake is assimilated by muscles, highlighting the preventive role of muscle mass in glycation stress [31]. Skeletal muscles are pivotal tissues for glucose homeostasis. In mice, diets rich in AGEs result in lower muscle mass and strength as well as decreases in fatigue resistance in comparison to low-AGE diets [26]. In vitro studies have revealed that cross-linking of collagen helps decrease sliding between fibers and the viscoelasticity of tendons and increases resistance to stress [26]. Therefore, a question arises regarding the relationship between AGEs and the stiffness of the stylomandibular ligament observed in both Eagle’s syndrome and temporomandibular dysfunction. In this context, the relevance of Kimmerle anomaly, comprising atlanto-occipital ligament rigidity, and its role in the aggravation of temporomandibular disorder could be of paramount importance [33].

And what about AGEs’ roles in fascia degeneration? Is there scientific evidence that fascial adhesions may result from the formation of cross-links? This facet seems to be crucial in the context of the pathology of myofascial pain and fascial restriction. Collagen glycation and cross-linking may affect the deep fascia [34]. Notably, “AGEs can build up in connective and fascial tissues and cause both inflammation and structural and functional disorders (…)” [35]. Thickening and fibrosis seem to be the consequences [34]. These findings alter structure–function properties, including decreases in tissue relaxation and accompanying increases in tissue yield stress [34]. This notion is gaining in importance with respect to temporomandibular disorders, especially myofascial pain with referral. Fascia’s indispensability with respect to the human body is demonstrated by the fact that about 10–15% of muscle force is anchored in the fascia, serving as evidence of extra-muscular transfer of myofascial force [36]. Interestingly, fascia can actively contract and consequently modulate musculoskeletal dynamics, and this capacity is unique [37].

Given that collagen turnover has half-lives of 4–12 months in the liver, 215 years in the intervertebral discs of elderly men [35], 100 years in cartilage, and 15 years in the skin, as well as 1% per day for the collagen of intramuscular fasciae [38], implementation of preventive measures against the degeneration of the structure of collagen seems to be paramount. Schleip et al. highlighted the phenomenon of mechanosensitive adaptation of fascial connective tissue with respect to Davis’s law, which applies to the adaptation of collagenous connective tissue morphology to mechanical loading [38]. They pointed out that both overstress and chronic underuse may trigger fibrotization. Insufficient challenges are associated with crystalline embrittlement of the ground substance and cross-links determined by advanced glycation end products. The tissue becomes brittle, demonstrating reduced flexibility and strength, and the perimysium undergoes sensitization [38]. From a clinical perspective, these findings are crucial in the context of temporomandibular myofascial pain; compensatory mechanisms in temporomandibular disorders, including hypertrophy of the masseter or/and temporal muscles; the etiology of dysfunctions conditioned by factors such as overuse/nonuse; and the physiology of pain. Interestingly, AGEs may contribute to hypothalamic inflammation and interrupt central control of energy balance [22]. There are no data on how these implications interfere with dysfunction of the hypothalamic–pituitary–adrenal axis in temporomandibular disorders such as bruxism. Thus, further research is needed.

Besides the endogenous formation of AGEs associated with normal metabolic processes, we can distinguish exogenous mechanisms/pathways conditioned by dietary behaviors [23]. It is widely accepted that cooking techniques characterized by high temperatures and low moisture conditions, including frying, grilling, roasting, broiling, and toasting, favor Maillard reaction pathways, thereby increasing the formation of AGEs [39,40]. Interestingly, manuka honey is a unique dietary source of methylglyoxal (MGO), a highly reactive dicarbonyl compound involved in the formation of AGEs. However, current clinical evidence does not demonstrate that moderate consumption of manuka honey significantly increases circulating AGE levels in healthy individuals [41].

Our previous studies revealed a potential link between jaw functional limitation scale (JFLS-20) scores and AGE levels [5]. The results suggested that decreased levels of AGEs may be connected with the global factor of JFLS-20, which may be related to the altered diet resulting from temporomandibular pain [5]. This may account for the lower AGE levels observed in the study group relative to the healthy controls. It is plausible that pain-induced dietary modifications influenced these findings.

Dietary AGEs are the consequence of non-enzymatic browning during the Maillard reaction, which occurs during the cooking, processing, and storage of food (e.g., in dry mixes or canned soups) [22,24]. Uncooked animal-derived foods contain AGEs, but their content increases with processing, especially thermal processing. Starting at 120–180°, the Maillard reaction is very rapid [24]. Grilling, frying, roasting, broiling, and searing favor AGE formation. These processes affect the flavor, color, and appearance of food; increase shelf-life; and eliminate food-borne diseases [24]. Heat-treated food contains 10–100 times more AGEs than untreated food [25]. Caramel is extremely dangerous in this regard. Dietary sources and endogenous synthesis account for the total load of AGEs in the body [25]. Under physiological conditions, about 30% of exogenous AGEs are excreted in urine, with the exception of individuals with kidney dysfunction and diabetes mellitus, in whom this value remains below 5% [25]. Anti-AGE strategies involve natural defense, lifestyle interventions (including with respect to dietary behaviors), and pharmacokinetic interventions and inhibitors, exemplified by metformin [25,42]. Accordingly, the “Eating the rainbow strategy” should be implemented [9,43]. Incorporation of antioxidant-rich dietary compounds, such as curcumin and quercetin, may represent a potential nutritional strategy for reducing AGE accumulation by limiting carbonyl stress and oxidative pathways involved in collagen glycation [44,45]. Regular physical activity may be an important modifiable factor influencing AGE homeostasis. Exercise-induced improvements in glucose metabolism and antioxidant capacity may attenuate carbonyl stress and reduce formation of new AGEs. However, recent evidence suggests that established AGE accumulation within long-lived proteins, including collagen, may not be fully reversible through exercise interventions alone [46,47].

Advanced oxidation protein product (AOPP) concentration is the next parameter in which significant differences might be observed between study and control groups. Our study results reveal lower AOPP concentrations in the study group relative to the healthy controls (_adj_ *p* = 0.0024) (Table 1). AOPPs were first described by Witko-Sarsat and are treated as a relevant biomarker of oxidative stress connected to tissue injury [48]. AOPPs arise from reactions between chlorinated compounds and proteins, resulting in the formation of dityrosine residues and subsequently protein cross-linking, aggregation, and precipitation [49].

After formation, AOPPs may affect tissue damage as a result of imbalance between antioxidant and pro-oxidant levels and ROS content in the body [50]. Besides oxidative dysregulation, AOPPs are involved in various other pathologies [49]. They accumulate in the human body during aging [51]. Increased AOPP levels are associated with non-communicable senescence-associated diseases such as atherosclerosis and cardiovascular disease, kidney disorders, uremia, inflammatory bowel syndrome, Alzheimer’s disease, osteoarthritis, osteoporosis, cancer, liver cirrhosis, and metabolic syndrome [36,50,52]. Elevated AOPP levels are associated with obesity and diabetes and correlate with glucose concentrations and age [48]. Increased AOPP levels are also observed in dementia [53]. The main pathomechanisms of AOPP activity include increased inflammation via macrophage mobilization; elevated IL-1, IL-6, and tumor necrosis factor (TNF-α) production; intensified formation of monocyte chemoattractant protein (MCP)-1 and stromal cell-derived factor (SDF-1) α; altered lipoproteins; and suppression of reverse cholesterol transfer [50]. AOPPs are implicated in cellular apoptosis, autophagy, inflammation, and senescence [51]. Analogously to advanced glycation end products, AOPPs interact via the receptor RAGE in endothelial cells, promoting its dysfunction [52]. Generally, AOPPs are associated with acute oxidative stress, while AGEs seem to be a biomarker of chronic damage [53].

AOPPs may play significant roles as novel markers of oxidative stress with respect to bones, serving as both biological drivers and marker of bone degradation [52]. There is some evidence that AOPPs are associated with increased osteoclastogenesis, osteoblast apoptosis, and impaired communication in osteocytes [51]. Furthermore, AOPPs may be implicated in bone–fat imbalance during skeletal aging [51]. AOPP accumulation tends to reduce bone formation and increase marrow adiposity [51]. As a consequence, AOPPs promote the transformation of osteoblasts into adipocytes and thus aging [51]. In addition, AOPPs contribute to anulus fibrosus cell senescence and accelerate intervertebral disc degeneration [54]. In the literature, there is no evidence of AOPPs playing a role in the degeneration of bones of the masticatory system, but it can be supposed that the processes above may imply dental bone augmentation or implantation processes. Furthermore, structural changes in the bone may modify their flexibility, which, apart from the aforementioned implant–prosthetic procedures, may reduce tolerance of functional deformation of the jaws during the use of prosthetic restorations. These changes do not remain neutral in the context of the functioning of the temporomandibular joint disc. Ege et al. found statistically significant increases in AOPP levels in patients with internal derangement of the temporomandibular joint relative to healthy controls [55]. On the basis of novel insights and the fact that metformin may inhibit the onset and progression of intervertebral disc degeneration [56], it can be assumed that it will also be useful in the prevention of oxidation-related temporomandibular disc disorders. Further research is needed.

AOPPs may be involved in pathophysiological processes in the central nervous system, especially hyperalgesia [57]. These molecules play a pivotal role in neuropathic pain through activating satellite glial cells and boosting inflammatory cytokine release via the RAGE/NF-κB signaling pathway [58]. Reducing AOPP accumulation may be an alleviating factor in neuropathic pain [58] and therefore orofacial pain. A study on rats revealed that advanced oxidative protein products may lead to pain hypersensitivity by initiating apoptosis of dorsal root ganglion neurons [59], which are extremely sensitive to ROS because of the low blood–nerve and perineural barriers in this area [59]. Notably, there are two main sources of cellular ROS: the nicotinamide adenine dinucleotide phosphate (NADPH) oxidase pathway and the mitochondrial respiratory cycle [59]. Excessive ROS formation affects mitochondrial membrane integrity and DNA through processes such as the loss of mitochondrial membrane potential and the triggering of DNA repair proteins [59]. Mitochondrial dysfunction and the mitogen-activated protein kinase pathway initiate cell apoptosis [59]. The dorsal horn of the spinal cord is similar to the trigeminal ganglia, to which the trigeminal nerve is attached [4]. Through this pathway, pain signals travel to the second-order neurons inside the brainstem [4]. There are no data in the literature on AOPPs’ potential contribution to orofacial pain through the trigeminal pathway, but it can be assumed that they do contribute in some way. More research is needed.

There is a relatively brief history of scientific investigation into protein oxidation [60]. There is a gap in the literature concerning the role of AOOPs in skeletal muscles. One study concerning sedentary behavior deserves special attention. It is widely known that changing one’s lifestyle from active to sedentary triggers rapid and marked limb muscle atrophy [61]. This changeover increases oxidative damage to skeletal fibers. In rats, seven days of a sedentary lifestyle led to a ~17% reduction in soleus muscle weight with respect to the bodyweight ratio. This phenomenon is associated with a simultaneous increase in oxidative damage biomarkers, including AOPPs [61]. A human study confirmed that a 6-month aerobic exercise could decrease levels of advanced oxidation protein products in a generally healthy population [62]. This finding support physical activity as a recommendation. Another potential implication concerns the use of metformin to reduce AOPP and AGE concentrations [25,49].

There are few studies on AOPPs’ roles in temporomandibular disorders. A study concerning orthodontic treatment with clear aligners and self-ligating brackets revealed an increase in AOPP levels after 90 days of treatment, with relatively stable total antioxidant capacity and myeloperoxidase content [63]. There is a gap in the literature regarding how prosthetic treatment influences oxidative biomarker profiles.

Another very interesting biomarker of oxidative stress is malondialdehyde (MDA). MDA is one of several molecules collectively quantified as thiobarbituric-acid-reactive substances (TBARS). It is the most reactive dialdehyde, so it easily combines with various functional groups of molecules such as proteins, lipoproteins, and DNA [64]. Due to its high reactivity, it responds very rapidly and does not require additional support such as heat or temperature [64]. Malondialdehyde can be produced through the oxidation of polyunsaturated fatty acids and via arachidonic acid metabolism [65]. Lipid peroxidation enables non-enzymatic formation of MDA. In turn, enzymatic processes entail synthesis of thromboxane A2, which arises through arachidonic acid metabolism under the influence of thromboxane A2 synthase and products of the breakdown of prostaglandin endoperoxide into 23-hydroxyheptadecatrienoate [65]. Lipid peroxidation—including MDA creation—occurs with respect to degradation products deriving from cellular damage. Arachidonic acid induces platelets to form a significant amount of MDA [65].

Shakouri et al. revealed that patients with general myofascial pain syndrome had higher plasma levels of MDA than healthy controls [66]. The authors highlighted oxidative stress’s pivotal role in pain pathology, including the fact that reactive oxygen/nitrogen species may directly and indirectly trigger sensitization and activation of nociceptors [66]. In skeletal muscles, ROS aggregation leads to myopathy and contractile impairment—two factors associated with myofascial pain syndrome [66].

Human studies on skeletal muscles have revealed that MDA may reflect active ROS status in injured tissue [67]. Lipid peroxidation is closely related to skeletal muscle damage, and MDA concentration is a relevant biomarker of chronic skeletal muscle injuries [67]. A study on rats showed similar trends [67,68]. Increased levels of MDA were observed in triceps after a grueling downhill run. It can be supposed that that muscular overuse/misuse may lead to changes in oxidative balance, and this process probably pertains to masticatory muscles as well. However, it should be emphasized that MDA concentrations can be decreased by vitamin E, which is an anti-lipid peroxidation agent [67].

Guan et al. showed that a group with injured skeletal muscle had almost double the level of MDA relative to a control group [67]. In our study, the TBARS concentration in saliva in the study group was three times higher than that in the healthy controls. Guan et al. proved that intermittent pressure, such as rolling manipulation, may lead to a decrease in MDA content in injured muscles [67]. It can be supposed that in patients with temporomandibular disorder, soft-tissue mobilization, including myofascial release [69], may benefit from an improvement in the dynamic balance of lipid peroxidation in the overused muscles.

Widyaadharma emphasized that excessive activity contributes to ROS formation [65]. Ongoing muscle contraction may aggravate metabolic stress and reduce blood flow, resulting in persistence of myofascial trigger points [65]. Oxidative stress elevates concentrations of free calcium and iron ions in cells, causing cell damage, including cell death [65]. Increased calcium levels lead to vasoconstriction and, as a consequence, ischemia, which, in turn, promotes ROS production [65]. This process results in endothelial damage, and it sustains further ischemia, which plays a crucial role in the pathophysiology of myofascial pain [65].

Excessive contraction is associated with stress that induces cytoskeletal microtubules to activate NADPH oxidase to produce ROS [65]. In skeletal muscles, isoforms 2 and 4 of NADPH play the main role in ROS formation during contraction [70]. They can be found in the plasma membrane, transvers striatum, and sarcoplasmic reticulum, affecting calcium regulation [70]. Calcium release may induce muscle contractions and consequently lead to the formation of myofascial trigger points [65]. Prolonged muscle contraction and associated ischemia/hypoxia, metabolic disorders, and cell stress contribute to excessive release of cytokines, myokines, and neurotransmitters, which seem to be paramount in the pathophysiology of myofascial pain [65]. Skeletal muscle damage induces excessive secretion of neuropeptides, cytokines, and inflammatory substances, including potassium, bradykinin, tumor necrosis factor TNF-α, interleukin 1 β, norepinephrine, protons, prostaglandins (especially PGE2), ATP, and substance P [65]. This activity could activate nociceptors in the muscles, resulting in calcitonin-gene-related peptide (CGRP) release [65]. CGRP may be involved in the increase in acetylcholine levels within trigger points and prolonged muscle contractions of local muscle fibers [65]. Sustained input within a myofascial trigger point may lead to apoptosis of inhibitory neurons at the segmental level and sensitize neurons of the dorsal horn, inducing hyperalgesia, allodynia, and permanent pain [65]. Critically, prolonged afferent nociceptive input from a myofascial trigger point stimulates and sensitizes the neurons in the dorsal horn, which, through the spinothalamic tract, induces the activity of higher brain centers, including the thalamus and limbic system, both of which are related to pain modulation, emotions, and body stress responses [65]. In addition, increased concentrations of neuropeptides, cytokines, potassium, bradykinins, TNF-α, and interleukins—all associated with MDA concentrations—may activate astrocyte changes in the central nervous system and thereby participate in hyperalgesia [65].

The next fascinating biomarker of oxidative stress is nitric oxide (NO). NO has a free -radical structure that makes it highly noxious [71]. An additional electron determines whether it is highly reactive. NO arises from the conversion of L-arginine into citrulline in a reaction supported by both NADPH and O_2_ as co-substrates, along with other cofactors [72]. As an endogenous molecule, at extremely low concentrations ranging from the pico- to nanomolar scale, NO is involved in physiological processes such as posttranslational modifications of proteins, mRNA translation, modulation of gene transcription, reproduction, apoptosis, vasodilation, immuno-responses, osteofunction, inflammation, angiogenesis, inhibition of platelet aggregation, cardiovascular homeostasis, biofilm inhibition, smooth-muscle relaxation, neurotransmission, and neuroprotection [71,73]. Decreased formation of NO leads to aging and cardiovascular diseases. Increased levels of NO result in oxidative stress, cytotoxicity, and associated neuroinflammation and neurodegenerative disorders [73]. NO has a very short half-life and can migrate limited distances before being oxidized [71]. It has a Janus face, with beneficial and harmful properties [72]. Nitric oxide plays a significant role in acute and chronic pain conditions at both central and peripheral levels [74]. It exerts anti- or pro-nociceptive or dual effects on the nervous system [74]. Freire et al. emphasized nitric oxide’s role in pain modulation in the spinal cord [72]. Similar to MDA, NO may play an important role in the pathophysiology of myofascial pain.

Freire et al. pointed out that repetitive stimulation arising from persistent tissue injury leads to spinal cord structures reorganization and the pain is exacerbated by nociceptive sensitization which has both central and peripheral components [72]. Chronic pain is associated with functional structural plasticity and rearrangement of the pain pathway [75].

Peripheral sensitization is associated with inflammatory mediators such as prostaglandin (PGE2), bradykinin, nerve growth factor (NGF), and NO at the site of damage [72]. This phenomenon aggravates the excitability of sensory terminals, resulting in localized tissue effects. Central sensitization arises from the repetitive activation of C-fibers and associated temporal summation of dorsal horn neuronal responses known as “windup” [72]. This sensitization is based on a mechanism similar to long-term potentiation (LTP). NO plays a pivotal role as a signaling molecule in LTP in the hippocampus and other cortical areas. The NO-related LTP of nociceptive pathways in the spinal cord is an essential pathway behind long-term central-pain sensitization [72].

In summary, “the management of myofascial pain can be optimally done by clinicians through blockage of each biomarker in a specific pathway” [65]. In light of the study we have conducted, our discussion reveals the putative roles of nutrition and eating behaviors, and their limitations, in oxidative–antioxidative imbalances as well as the pathophysiology and etiology of TMDs, including myofascial pain with referral. Using the literature as a basis, we addressed the risk of lifestyle- and diet-related diseases associated with oxidative stress. Subsequent investigations in this domain ought to encompass a rigorous analysis of patients’ nutritional patterns and degrees of physical activity.

The favorably low levels of protein glycation and oxidation (as indicated by reductions in quantities of AGEs and AOPPs) may reflect effective dietary mitigation of glyco-oxidative stress in our study group, highlighting the protective effect of targeted carbohydrate management and thermal processing control on systemic protein status. Nevertheless, the dietary modifications observed were putatively induced by temporomandibular myofascial pain with referral, as we demonstrated using JFLS-20 in a previous publication [5]. Concurrent elevations in levels of TBARS and total NO pointed to ongoing lipid peroxidation indicated by oxidative degradation of unsaturated fatty acids. Vitamins C and E and polyphenols may help mitigate this targeted oxidative stress. In addition, rigorous quality control measures and appropriate cold-chain storage of dietary fats and omega-3 fatty acid supplements would be essential for the consumption of pre-oxidized lipids. Antioxidant-rich culinary herbs such as rosemary, oregano, and turmeric may further attenuate oxidative processes.

This study’s main limitation is the gender disparity between groups, which featured a strong predominance of female participants. Research with a more balanced sample is required. The lack of detailed nutritional profiles for both the patients and control group limits interpretation of the results.

Another limitation of this study is our sequential recruitment and analysis of the study group (recruited in 2016–2017 and analyzed in 2024) and control group (recruited in 2023 and analyzed in 2023). Although all assays were performed using identical equipment and by the same investigator using standardized reagents from the same manufacturer, formal long-term stability validation under specific storage conditions was not performed prior to analysis. However, all samples were constantly maintained at −80° C without freeze–thaw cycles to preserve sample integrity to the greatest extent possible. Consequently, we cannot entirely exclude potential confounding from storage duration, inter-lot reagent variations, or subtle instrumental drift over time.

A formal a priori sample-size calculation was not performed prior to study initiation, reflecting the exploratory nature of this investigation. Although the size of our cohort was sufficient to reveal adjusted differences in several biomarkers, statistical power may have been limited in the gender-stratified analyses, especially within the male subgroup. Consequently, adjusted absolute effect sizes (β), alongside their 95% confidence intervals, were provided. Nonetheless, our findings should be regarded as hypothesis-generating, and future research with larger, formally powered study groups should be conducted to validate these observations.

Our highly stringent inclusion criteria prioritized internal validity to isolate biomarker alterations from potential biomechanical confounders such as incomplete natural dentition, malocclusion, and missing-canine guidance. Selecting patients with high pain intensity (VAS ≥ 8) ensured that we obtained pronounced, detectable biological signals, minimizing borderline variability in biomarker expression. As pharmacological treatments, medical or dental procedures, and underlying chronic conditions may disrupt salivary oxidative-antioxidant balance, these factors were identified as potential confounders and used as strict exclusion criteria. However, the abovementioned factors restrict our study’s external validity, and the findings cannot be directly extrapolated to the broader, more anatomically and clinically heterogeneous TMD patient population.

Additionally, the assessment of salivary AGEs was based on total fluorescence, which is relatively non-specific and reflects the cumulation pool of glycation products rather than separated molecular species. Although this method is suitable for screening purposes, subsequent investigations should incorporate more advanced and specific analytical approaches.

A notable limitation concerns the evaluation of lipid peroxidation using the thiobarbituric acid (TBA) method. TBA may cross-react with aldehydes and non-lipid compounds present in saliva, which are collectively referred to as thiobarbituric-acid-reactive substances (TBARS). Although the TBA assay is a widely accepted and valuable indicator of oxidative stress, it offers limited analytical specificity and may overestimate the absolute concentration of malondialdehyde (MDA) relative to advanced analytical methods such as high-performance liquid chromatography (HPLC) and mass spectrometry.

The next limitation involves the fact that salivary nitric oxide production was evaluated according to its stable end-products rather than via direct radical capture. Due to this compound’s reactive nature and fleeting biological half-life, direct analytical determination in biological samples is practically unfeasible. Consequently, the assay quantified its stable metabolites: nitrate/nitrite. Although widely accepted, this indirect evaluation may not perfectly reflect nitric oxide release.

## 5. Conclusions

1. Myofascial pain with referral is independently associated with oxidative imbalance, highlighting the relevance of TBARS and total NO as promising severity biomarkers.
2. The unexpected decrease in AGEs and AOPPs requires standardized studies to confirm diagnostic utility.
3. This study’s findings highlight the necessity of future research exploring dietary interventions and antioxidant therapy as potential mitigating factors against oxidative stress and novel strategies for alleviating temporomandibular myofascial pain.

## Figures and Tables

**Table 1:** Salivary concentrations of oxidative and nitrosative stress biomarkers and results of multiple linear regression, including models adjusted for age, sex, and salivary flow rate across the study and control groups and the sex-stratified subgroups.

Biomarkers	Units	Study Group n = 44			Control Group n = 44			Multiple Linear Regression			
		Mean	±SD	Median	Mean	±SD	Median	Estimate β [95% CI] ^a^		*p*-Value	_adj_ *p* ^b^
AGE	AFU/mg protein	17.93	16.58	15.01	21.11	21.60	12.20	−5.35	[95% CI: −14.34, 3.644]	0.2403	0.2403
AOPP	nmol/mg protein	217.6	215.8	151.1	994.7	1319	505.3	−755.7	[95% CI: −1228, −283.5]	0.0021	0.0028
TBARS	μmol/mg protein	7.777	7.750	6.078	2.587	2.764	1.486	4.78	[95% CI: 2.020, 7.541]	0.0009	0.0018
Total NO	μmol/mg protein	162.1	131.5	138.6	29.99	25.66	23.82	128.4	[95% CI: 82.70, 174.1]	<0.0001	<0.0004
		Study (Women) n = 32			Control (Women) n = 12						
AGE	AFU/mg protein	16.56	6.407	16.04	24.56	23.34	13.00	−11.17	[95% CI: −20.30, 2.046]	0.0174	0.0174
AOPP	nmol/mg protein	239.7	234.5	152.8	940.0	1155	326.9	−754.5	[95% CI: −1215, −294.4]	0.0018	0.0024
TBARS	μmol/mg protein	6.871	3.583	6.184	2.912	3.205	1.781	3.36	[95% CI: 1.317, 5.393]	0.0018	0.0024
Total NO	μmol/mg protein	168.7	144.6	140.0	32.67	26.81	27.63	123.7	[95% CI: 55.91, 191.5]	0.0006	0.0024
		Study (Men) n = 12			Control (Men) n = 22						
AGE	AFU/mg protein	21.58	30.64	13.52	17.66	19.64	9.954	3.79	[95% CI: −18.06, 25.64]	0.7258	0.7258
AOPP	nmol/mg protein	141.6	111.2	128.0	1055	1507	379.4	688.5	[95% CI: −2277, 559.1]	0.2238	0.2984
TBARS	μmol/mg protein	10.19	13.78	5.932	2.292	2.331	1.405	3.6	[95% CI: 0.1730, 15.22]	0.0453	0.0906
Total NO	μmol/mg protein	139.5	71.75	109.2	27.19	24.74	19.22	116.2	[95% CI: 70.66, 161.7]	<0.0001	<0.0004

^a^ The adjusted effect size reflects the unstandardized linear regression coefficient (*β*) with 95% confidence intervals (95% CIs), adjusted to age, gender, and salivary flow rate. The control group serves as a reference. ^b^ *p*_adj_—*p*-value adjusted for multiple-hypothesis testing across the four salivary biomarkers using the Benjamini–Hochberg false-discovery-rate procedure (Q = 0.05).

## Data Availability

This article contains all the data used to support its findings.

## Abbreviations

The following abbreviations are used in this manuscript:

TMDs	Temporomandibular Disorders
ROS	Reactive Oxygen Species
DC/TMD	Diagnostic Criteria for Temporomandibular Disorders
VAS	Visual Analogue Scale
AGEs	Advanced Glycation End Products
AOPPs	Advanced Oxidation Protein Products
MDA	Malondialdehyde
NO	Nitric Oxide
TBARS	Thiobarbituric-Acid-Reactive Substances
